# Identification of predictive markers of the therapeutic effect of eribulin chemotherapy for locally advanced or metastatic breast cancer

**DOI:** 10.1186/s12885-017-3598-5

**Published:** 2017-08-31

**Authors:** Shinichiro Kashiwagi, Wakaba Fukushima, Yuka Asano, Wataru Goto, Koji Takada, Satoru Noda, Tsutomu Takashima, Naoyoshi Onoda, Masahiko Ohsawa, Kosei Hirakawa, Masaichi Ohira

**Affiliations:** 1 Department of Surgical Oncology, Osaka City University Graduate School of Medicine, 1-4-3 Asahi-machi, Abeno-ku, Osaka, 545-8585 Japan; 2 Department of Public Health, Osaka City University Graduate School of Medicine, 1-4-3 Asahi-machi, Abeno-ku, Osaka, 545-8585 Japan; 3 Department of Diagnostic Pathology, Osaka City University Graduate School of Medicine, 1-4-3 Asahi-machi, Abeno-ku, Osaka, 545-8585 Japan

**Keywords:** Triple-negative breast cancer, TLE3, β-tubulin class III, GSTP1, Microtubule dynamics inhibitor

## Abstract

**Background:**

The recently developed reagent, eribulin mesylate (eribulin), is a microtubule dynamics inhibitor with a mechanism of action that differs from those of taxanes and vinca alkaloids. This drug is considered to be a promising chemotherapeutic agent for the treatment of locally advanced or metastatic breast cancer (MBC). In this study, we investigated if variables such as tumor expression of β-tubulin class III, glutathione S-transferase pi (GSTP) 1 or transducin-like enhancer of split (TLE) 3 might act as predictive factors on the therapeutic effect of eribulin chemotherapy.

**Methods:**

The subjects included 52 patients with MBC who underwent chemotherapy with eribulin. The expression levels of Estrogen receptor (ER), progesterone receptor (PgR), human epidermal growth factor receptor (HER) 2, Ki67, β-tubulin class III, GSTP-1 and TLE-3 were evaluated using immunostaining employing needle biopsy specimens.

**Results:**

Patients with TLE3-negative tumors displayed significantly poorer outcomes regarding progression-free survival than patients with TLE3-positive tumors when prognosis within the group of patients with triple-negative breast cancer (TNBC) lesions was analyzed (*p* = 0.011, log-rank). In contrast, no such difference in prognosis was found in a comparison of TLE-3 positive/negative patients in the group of all patients (*p* = 0.433, log-rank) or of patients with non-TNBC lesions (*p* = 0.659, log-rank). Based on a univariate analysis of 22 TNBC cases, a better progression-free survival correlated significantly with a positive TLE3 expression in the tumor (*p* = 0.025). A multivariate logistic regression analysis including 22 patients with TNBC also showed that a positive TLE3 expression significantly correlated with a better progression-free survival (*p* = 0.037).

**Conclusions:**

Our findings suggest that TLE3 is a useful marker for predicting the therapeutic effect of eribulin chemotherapy for TNBC.

**Electronic supplementary material:**

The online version of this article (10.1186/s12885-017-3598-5) contains supplementary material, which is available to authorized users.

## Background

Triple-negative breast cancer (TNBC), which is characterized by negativity for Estrogen receptor (ER), progesterone receptor (PgR), and human epidermal growth factor receptor type (HER) 2, is a high-risk breast cancer that lacks specific targets for treatment selection [1–8]. TNBC involves many cases in which a satisfactory effect of chemotherapy is not observed. However, a remarkable effect is occasionally in some cases; therefore, accurate prediction of the therapeutic effect would not only allow direct interpretation of the effect of treatment but would also be beneficial for preventing adverse events due to invalid treatment. Consequently, it is crucial that markers capable of predicting the therapeutic effect of chemotherapeutic agents be identified, and that tumors with intrinsic biological subtypes are stratified.

Taxane is a key drug in chemotherapy regimens for metastatic breast cancer (MBC). The recently developed reagent, eribulin, is a microtubule dynamics inhibitor with an action mechanism that differs from those of taxane and vinca alkaloid [9–11]. This agent binds to the polymerized region of microtubules with high affinity, preventing the microtubules from extending and thus halting cell cycle arrest in the G2 phase [12, 13]. Eribulin treatment was recently reported to achieve prolonged overall survival in patients with MBC in a phase III clinical trial [14]; thus, this drug is considered to be a promising chemotherapeutic agent for the treatment of MBC. Curing MBC is often difficult, except a few cases; therefore, the objective of treatment is commonly the prolongation of survival, with the aim of maintaining the quality of life (QOL). Therefore, it is essential to both minimize the rate of adverse events accompanying treatment and to improve the associated symptoms of tumor regression. Moreover, breast cancer is a very diverse disease regarding tumor biology, as stated above, with wide variation among individuals regarding sensitivity to anticancer drugs. Accordingly, to achieve maximum results from chemotherapy, it is necessary to predict the efficacy of treatment and select the optimum pharmacotherapy according to the characteristics of both the patient and the tumor.

Although eribulin has a pharmacological effect due to its effect on microtubule formation in the same manner as a conventional taxane, it has been shown to display no cross-resistance due to its mechanism of action, which differs from that of other taxanes [15]. Moreover, as a result of its excellent efficacy against TNBC, as demonstrated in a subanalysis of phase III clinical trial, eribulin is expected to become a key drug for managing patients with TNBC in the future.

In this study, we investigated if factors such as transducin-like enhancer of split (TLE) 3 [16–18], β-tubulin class III [19–22] and glutathione S-transferase pi (GSTP) 1 [23, 24], which have previously been reported to be predictive factors of the therapeutic effect of taxanes, might act as predictive factors regarding the therapeutic effect of eribulin chemotherapy, with the aim of identifying possible biomarkers for predicting the efficacy of eribulin.

## Methods

### Patient background

The subjects included 52 patients with inoperable or metastasis/recurrent breast cancer who underwent chemotherapy using eribulin from August 2011 to June 2013 at our institute. Our previous reports have also used the same patient population and the present study, but it was the study of the significance of tumor-infiltrating lymphocytes [25]. The median follow-up time was 431 days (range, 50–650 days). The overall response rate (ORR), clinical benefit rate (CBR), disease control rate (DCR), overall survival (OS), time to treatment failure (TTF) and progression-free survival (PFS) were calculated regarding the efficacy of this regimen. Additionally, based on the immunohistochemical expression of ER, PgR, HER2 and Ki67, the tumors were categorized into immunophenotypes of luminal A (ER+ and/or PgR+, HER2-, Ki67-low), luminal B (ER+ and/or PgR+, HER2+) (ER+ and/or PgR+, HER2-, Ki67-high), HER2-enriched (ER-, PgR-, and HER2+), and TNBC (negative for ER, PgR and HER2).

Regarding the outline of the chemotherapy regimen, one course of treatment consisted of 21 days (three weeks). Eribulin mesylate (1.4 mg/m^2^) was intravenously administered on days 1 and 8, after which a withdrawal period was continued to day 21. This protocol was repeated until progressive disease (PD) was detected or a severe adverse event requiring the discontinuation of the scheduled chemotherapy was noted. The chemotherapy was administered on an outpatient basis in all cases. The antitumor effect was evaluated based on the criterion for therapeutic effects conforming to the RECIST criteria (Response Evaluation Criteria in Solid Tumors) version 1.1 [26, 27].

The morphology of the tumor, including the histological tissue type, nucleus grade, etc., was evaluated using conventional hematoxylin and eosin (HE) staining, and the expression levels of ER, PgR, HER2, Ki67, β-tubulin class III, GSTP1 and TLE3 were evaluated using immunostaining employing a needle biopsy specimen obtained prior to the start of chemotherapy with eribulin. The pathological diagnosis was made by several experienced pathologists specialized in cancer. This research conformed to the provisions of the Declaration of Helsinki in 1995. All patients were informed of the investigational nature of this study and provided their written informed consent. The study protocol was approved by the Ethics Committee of Osaka City University (#926).

TTF was evaluated on a daily basis and was set as the period from the date of treatment commencement to cancellation for any reason, including disease aggravation, treatment toxicity, and death. OS was evaluated on a daily basis and was set as the period from the date of treatment commencement to death. PFS was evaluated on a daily basis and was set as the period from the date of treatment commencement to the earlier of the date of death or confirmation of PD.

### Immunohistochemistry

Immunohistochemical studies were performed as previously described [28, 29]. The tumor specimens were fixed in 10% formaldehyde solution and embedded in paraffin, after which they were cut into 4-μm-thick sections and mounted on glass slides. The slides were deparaffinized in xylene and heated for 20 min at 105 °C and 0.4 kg/m^2^ using an autoclave in Target Retrieval Solution (Dako, Carpinteria, California, USA). The specimens were then incubated with 3% hydrogen peroxide in methanol for 15 min to block the endogenous peroxidase activity and were subsequently incubated with 10% normal goat or rabbit serum to block nonspecific reactions.

Primary monoclonal antibodies directed against ER (clone 1D5, dilution 1:80; Dako), PgR (clone PgR636, dilution 1:100; Dako), HER2 (HercepTest™; Dako), Ki67 (clone MIB-1, dilution 1:00; Dako), β-tubulin class III (clone SDL.3D10, dilution 1:400; Sigma-Aldrich), GSTP1 (clone 3F2, dilution 1:800; Cell Signaling) and TLE3 (clone S0733, dilution 1:2000; Clarient) were used. The tissue sections were incubated with each antibody for 70 min at room temperature or overnight at 4 °C and were then incubated with horseradish peroxidase-conjugated anti-rabbit or anti-mouse Ig polymer as a secondary antibody (HISTOFINE (PO)™ kit; Nichirei, Tokyo). The slides were subsequently treated with streptavidin–peroxidase reagent and incubated in phosphate-buffered saline–diaminobenzidine and 1% hydrogen peroxide (*v*/v), followed by counterstaining with Mayer’s hematoxylin. Positive and negative controls for each marker were used according to the supplier’s data sheet.

### Immunohistochemical scoring

**Fig. 1:**
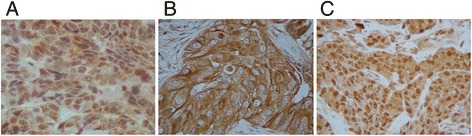
Immunohistochemical determination of TLE3, β-tubulin class III and GSTP1. Representative immunohistochemical staining of the indicated proteins in tumor tissue is shown (×400). Only nuclear staining was considered specific for TLE3 **a**. Cytoplasmic staining by β-tubulin class III **b** and GTSP1 **c** antibodies was observed in the cancer cells

The cut-off value for ER and PgR positivity was ≥1% positive tumor cells with nuclear staining. HER2 expression was graded according to the accepted grading system as 0, 1+, 2+ or 3+. The following criteria were used for scoring: 0, no reactivity or membranous reactivity in less than 10% of cells; 1+, faint/barely perceptible membranous reactivity in 10% of cells or higher reactivity in only a part of the cell membrane; 2+, weak to moderate complete or basolateral membranous reactivity in 10% of tumor cells or higher and/or strong complete or basolateral membranous reactivity in 10% or higher in 30% or lower of tumor cells; 3+, strong complete or basolateral membranous reactivity in more than 30% of tumor cells. HER2 was considered to be positive if the grade of immunostaining was 3+, or a 2+ result showed gene amplification via fluorescent in situ hybridization (FISH). In the FISH analyses, each copy of the HER2 gene and its centromere 17 (CEP17) reference were counted. The interpretation followed the criteria of the ASCO/CAP guidelines for HER2 IHC classification for breast cancer, i.e., positive if the HER2/CEP17 ratio was higher than 2.0. A Ki67-labelling index of ≥14% was classified as positive. Only nuclear staining was considered distinct for TLE3. Cytoplasmic staining by β-tubulin class III and GTSP1 antibodies was observed in the cancer cells. The TLE3 and GSTP1 expression levels were semi-quantitatively analyzed according to the percentage of cells showing specific staining: 0, 0–10%; 1+, 10–30%; 2+, 30–70%; 3+, >70%. TLE3 expression was considered positive for scores of ≥2 and negative for scores of ≤1 **(**Fig. 1a **)** [17, 18]. GSTP1 expression was considered positive for scores of ≥1 and negative for a score of 0 **(**Fig. 1c **)** [23]. Tumor cells were acquired concerning the normally strong level of β-tubulin class III cytoplasmic staining within endothelial cells or nerves. Tumor cells that stained with at least equal intensity to the endothelial cells or nerves were considered to be positive. To determine the correlations with patient outcomes, the samples were scored as follows: (no staining), 1 (<50% positive cells) or 2 (≥50% positive cells) **(**Fig. 1b **)** [20–22].

### Statistical analysis

Continuous data are reported as the median (range). Statistical analysis was performed using the SPSS® version 13.0 statistical software package (IBM, Armonk, New York, USA). The associations between the expression of TLE3, β-tubulin class III or GSTP1 and the clinicopathological parameters were analyzed using the chi-squared test and chi-square test (or Fisher’s exact test when necessary) for trends, as appropriate. The Kaplan-Meier method was used to estimate the values of OS, TTF, and PFS. The OS, TTF, and PFS values were compared using the log-rank test. Events for the calculation of PFS induced disease progression. The Cox proportional hazards model was used to compute univariate and multivariate hazard ratios for the study parameters with 95% confidence intervals (CI) and was used in a backward stepwise method for variate selection in multivariate analysis. In all of the tests, a *p*-value of less than 0.05 was considered statistically significant. Cut-off values for different biomarkers included in this study was chosen before statistical analysis.

## Results

### Clinical effects of eribulin chemotherapy

The subjects included 52 patients who underwent chemotherapy using eribulin against inoperable or metastasis/recurrent breast cancer. The gender was female in all cases, with a median age of 63.5 ± 12.7 years. Regarding the line of administration (excluding adjuvant therapy), the average number of chemotherapeutic regimens that had been undertaken before eribulin administration was 2.4 ± 2.3, including 19 opportunities as first-line therapy. A total of 39 patients (75.0%) were suffering from visceral metastases at the administration, and the lesions in 14 cases were considered to be life-threatening. The site of metastasis included, in decreasing order: lung, 19 cases (36.5%); bone, 19 cases (36.5%); liver, 18 cases (34.6%) (Table 1).

**Table 1:** Demographical data of 52 patients with eribulin chemotherapy for locally advanced or metastatic breast cancer

Parameters (*n* = 52)	Number of patients (%)
Age (years old)	63.5 ± 12.7
Degree of progress	
Locally advanced / Visceral metastases	13 (25.0%) / 39 (75.0%)
Site of metastases	
Lung / Bone / Liver	19 (36.5%) / 19 (36.5%) / 18 (34.6. %)
Life threatening condition	
Life threatening / non- Life threatening	14 (26.9%) / 38 (73.1%)
Nuclear grade	
1 / 2 / 3	13 (25.0%) / 20 (38.5%) / 19 (36.5%)
Estrogen receptor	
Negative / Positive	25 (48.1%) / 27 (51.9%)
Progesterone receptor	
Negative / Positive	32 (61.5%) / 20 (38.5%)
HER2	
Negative / Positive	47 (90.4%) / 5 (9.6%)
Ki67	
Negative / Positive	26 (50.0%) / 26 (50.0%)
Intrinsic subtype	
Luminal A/Luminal B/Luminal HER2/HER2 enriched/TNBC	12 (23.1%) / 13 (15.0%) / 2 (3.8%) / 3 (5.8%) / 22 (42.3%)

*HER2* human epidermal growth factor receptor 2, *TNBC* triple-negative breast cancer

**Fig. 2:**
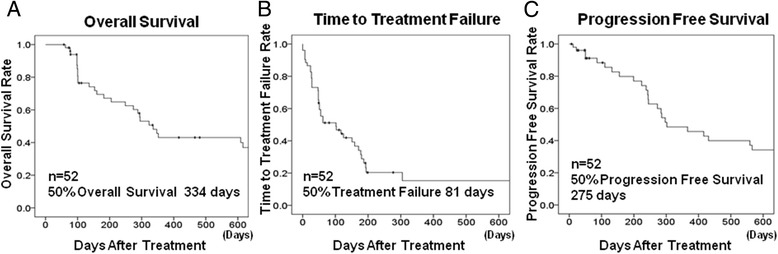
Clinical effects of eribulin chemotherapy. Kaplan-Meier curves of the indicated clinical effects of eribulin chemotherapy are shown. The clinical effects were as follows: median overall survival (OS) = 334 days **a**; median time to treatment failure (TTF) = 81 days **b**; and median progression-free survival (PFS) = 275 days **c**

The clinical effects of eribulin were as follows: ORR, 34.6% (18/52); CBR, 44.2% (23/52); DCR, 51.9% (27/52); median OS, 334 days; median TTF, 81 days; and median PFS, 275 days (Fig. 2a, b, c). The distribution of the intrinsic subtype classification was as follows: Luminal A, 12 cases (23.1%); Luminal B, 13 cases (15.0%); Luminal HER2, 2 cases (3.8%); HER2 enriched, 3 cases (5.8%) (non-TNBC 30 cases, 57.7%); and TNBC, 22 cases (42.3%). In investigation according to the intrinsic subtype, ORR was found to be 40.0% (12/30) in the non-TNBC cases and 27.3% (6/22) in the TNBC cases (Table 2).

**Table 2:** Clinical effects of eribulin chemotherapy in breast cancer subtype

	All breast cancer (*n* = 52)	Intrinsic subtype	
		non-Triple-negative (*n* = 30, 57.7%)	Triple-negative (*n* = 22, 42.3%)
ORR; Objective Response Rate	18 (34.6%)	12 (40.0%)	6 (27.3%)
CBR; Clinical Benefit Response	23 (44.2%)	15 (50.0%)	8 (36.4%)
DCR; Disease Control Rate	27 (51.9%)	18 (60.0%)	9 (40.9%)
CR; Complete Response	1 (1.9%)	1 (3.3%)	0 (0.0%)
PR; Partial Response	17 (32.7%)	11 (36.7%)	6 (27.3%)
SD; Stable Disease >24wks	5 (9.6%)	3 (10.0%)	2 (9.1%)
SD; Stable Disease	4 (7.7%)	3 (10.0%)	1 (4.5%)
PD; Progressive Disease	20 (38.5%)	7 (23.3%)	13 (59.1%)
NE; Not Evaluable	5 (9.6%)	5 (16.7%)	0 (0.0%)

### Expression of markers in patients with locally advanced or metastatic breast cancer

TLE3, β-tubulin class III, and GSTP1 were expressed in 24 cases (46.2%), 21 cases (40.4%) and 24 cases (46.2%), respectively, among the 52 patients investigated. The expression of TLE3 was found significantly more frequently in the TNBC lesions than in the non-TNBC lesions (*p* = 0.030). However, no significant differences were found between the expression of either TLE3, β-tubulin class III or GSTP1 in the tumors and the clinicopathological background factors of the patients or tumors **(**Table 3 **)**. In a multivariate analysis including TLE3 and Ki67, no biomarkers useful for predicting the efficacy of eribulin in cases of MBC were found (Additional file 1).

**Table 3:** Correlation between clinicopathological features and β-tubulin class III, GSTP1, and TLE3 expression in 52 locally advanced or metastatic breast cancer

Parameters		β-tubulin class III		*p* value	GSTP1		*p* value	TLE3		*p* value
		Positive (*n* = 21)	Negative (*n* = 31)		Positive (*n* = 24)	Negative (*n* = 28)		Positive (*n* = 24)	Negative (*n* = 28)	
HR and HER2 status										
TNBC		7 (33.3%)	15 (48.4%)	0.281	13 (54.2%)	9 (32.1%)	0.109	14 (58.3%)	8 (28.6%)	0.030
non-TNBC		14 (66.7%)	16 (51.6%)		11 (45.8%)	19 (67.9%)		10 (41.7%)	20 (71.4%)	
Age at operation										
≤63		9 (42.9%)	17 (54.8%)	0.397	10 (41.7%)	16 (57.1%)	0.266	15 (62.5%)	11 (39.3%)	0.095
>63		12 (57.1%)	14 (45.2%)		14 (58.3%)	12 (42.9%)		9 (37.5%)	17 (60.7%)	
Degree of progress										
Locally advanced		6 (28.6%)	7 (22.6%)	0.624	4 (16.7%)	9 (32.1%)	0.168	6 (25.0%)	7 (25.0%)	1.000
Visceral metastases		15 (71.4%)	24 (77.4%)		20 (83.3%)	19 (67.9%)		18 (75.0%)	21 (75.0%)	
Life threatening condition										
non- Life threatening		16 (76.2%)	22 (71.0%)	0.677	15 (62.5%)	23 (82.1%)	0.111	15 (62.5%)	23 (82.1%)	0.111
Life threatening		5 (23.8%)	9 (29.0%)		9 (37.5%)	5 (17.9%)		9 (37.5%)	5 (17.9%)	
Nuclear grade										
1, 2		13 (61.9%)	20 (64.5%)	0.848	14 (58.3%)	19 (67.9%)	0.477	13 (54.2%)	20 (71.4%)	0.198
3		8 (38.1%)	11 (35.5%)		10 (41.7%)	9 (32.1%)		11 (45.8%)	8 (28.6%)	
Estrogen receptor										
Negative		8 (38.1%)	17 (54.8%)	0.236	14 (58.3%)	11 (39.3%)	0.171	15 (62.5%)	10 (35.7%)	0.054
Positive		13 (61.9%)	14 (45.2%)		10 (41.7%)	17 (60.7%)		9 (37.5%)	18 (64.3%)	
Progesterone receptor										
Negative		12 (57.1%)	20 (64.5%)		16 (66.7%)	16 (57.1%)		18 (75.0%)	14 (50.0%)	
Positive		9 (42.9%)	11 (35.5%)	0.592	8 (35.3%)	12 (42.9%)	0.482	6 (25.0%)	14 (50.0%)	0.065
HER2										
Negative		18 (85.7%)	29 (93.5%)	0.317	23 (95.8%)	24 (85.7%)	0.227	23 (95.8%)	24 (85.7%)	0.227
Positive		3 (14.3%)	2 (6.5%)		1 (4.2%)	4 (14.3%)		1 (4.2%)	4 (14.3%)	
Ki67										
Negative		11 (52.4%)	15 (48.4%)	0.777	14 (58.3%)	12 (42.9%)	0.266	12 (50.0%)	14 (50.0%)	1.000
Positive		10 (47.6%)	16 (51.6%)		10 (41.7%)	16 (57.1%)		12 (50.0%)	14 (50.0%)	

*GSTP 1* glutathione S-transferase pi 1, *TLE3* transducin-like enhancer of split 3, *HR* hormone receptor, *HER2* human epidermal growth factor receptor 2, *TNBC* triple-negative breast cancer

### TLE3 expression in patients with triple-negative breast cancer

**Fig. 3:**
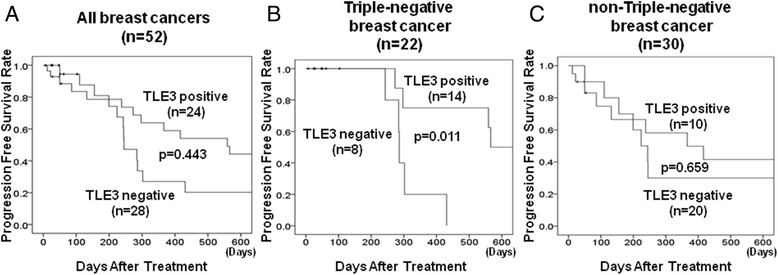
Progression-free survival of patients with MBC based on TLE3 expression. Kaplan-Meier curves of progression free survival of all patients **a**, of patients with TNBC lesions **b** and of patients with non-TNBC lesions **c** according to TLE3 expression. Patients with TNBC lesions that had TLE3-negative tumors experienced significantly poorer prognosis in terms of progression-free survival than those with TLE3-positive tumors (*p* = 0.011) **b**. In contrast, no significant differences were found in the progression free survival of TLE3-positive/−negative groups within all patients (*p* = 0.433) **a** or within patients with non-TNBC lesions (*p* = 0.659) **c**

**Fig. 4:**
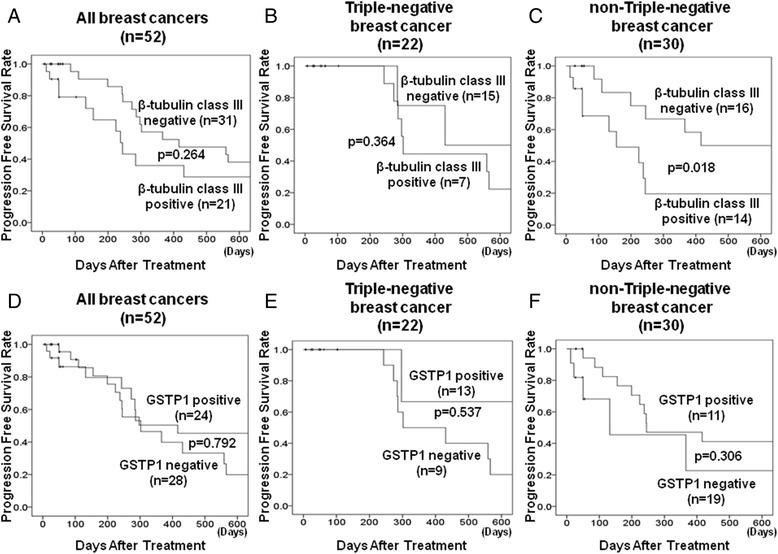
PFS of patients with MBC based on β-tubulin class III and GSTP1 expression. No significant differences were observed in β-tubulin class III or GSTP1 expression in the MBC, TNBC and non-TNBC groups **a**, **b**, **d**, **e**, **f**, with the exception of the β-tubulin class III expression in the non-TNBC group (*p* = 0.018) **c**

TNBC, TLE3, β-tubulin class III and GSTP1 were expressed in 14 cases (63.6%), seven cases (31.8%) and 13 cases (59.1%), respectively, among 22 tumors showing characteristics of TNBC. When the clinicopathological background characteristics and expression of each factor were investigated, no factors are significantly affecting the expression levels of these three factors were identified (Table 4). However, patients with TLE3-negative tumors displayed significantly poorer outcomes in terms of PFS than patients with TLE3-positive tumors when the prognosis of patients with TNBC lesions was analyzed (*p* = 0.011, log-rank) (Fig. 3b) In contrast, no significant differences were found between TLE3-negative/positive patients when the prognosis of all patients (*p* = 0.433, log-rank) or of patients with non-TNBC lesions (*p* = 0.659, log-rank) was investigated (Fig. 3a, c). On the other hand, no significant differences were observed in β-tubulin class III or GSTP1 expression among the MBC, TNBC or non-TNBC groups (Fig. 4a, b, d, e, f), with the exception of β-tubulin class III expression in the non-TNBC group (*p* = 0.018, log-rank) (Fig. 4c).

**Table 4:** Correlation between clinicopathological features and β-tubulin class III, GSTP1, and TLE3 expression in 22 triple-negative breast cancers

Parameters		β-tubulin class III		*p* value	GSTP1		*p* value	TLE3		*p* value
		Positive (*n* = 7)	Negative (*n* = 15)		Positive (*n* = 13)	Negative (*n* = 9)		Positive (*n* = 14)	Negative (*n* = 8)	
Age at operation										
≤63		4 (57.1%)	8 (53.3%)	0.616	6 (46.2%)	6 (66.7%)	0.305	9 (64.3%)	3 (37.5%)	0.221
>63		3 (42.9%)	7 (46.7%)		7 (53.8%)	3 (33.3%)		5 (35.7%)	5 (62.5%)	
Degree of progress										
Locally advanced		0 (0.0%)	6 (40.0%)	0.067	3 (23.1%)	3 (33.3%)	0.477	2 (14.3%)	4 (50.0%)	0.096
Visceral metastases		7 (100.0%)	9 (60.0%)		10 (76.9%)	6 (66.7%)		12 (85.7%)	4 (50.0%)	
Life threatening condition										
non- Life threatening		4 (57.1%)	5 (33.3%)	0.276	5 (38.5%)	4 (44.4%)	0.561	8 (57.1%)	1 (12.5%)	0.052
Life threatening		3 (42.9%)	10 (66.7%)		8 (61.5%)	5 (55.6%)		6 (42.9%)	7 (87.5%)	
Nuclear grade										
1, 2		1 (14.3%)	4 (26.7%)	0.477	4 (30.8%)	1 (11.1%)	0.293	4 (28.6%)	1 (12.5%)	0.380
3		6 (85.7%)	11 (73.3%)		9 (69.2%)	8 (88.9%)		10 (71.4%)	7 (87.5%)	
Ki67										
Negative		3 (42.9%)	8 (53.3%)	0.500	7 (53.8%)	4 (44.4%)	0.500	8 (57.1%)	3 (37.5%)	0.330
Positive		4 (57.1%)	7 (46.7%)		6 (46.2%)	5 (55.6%)		6 (42.9%)	5 (62.5%)	

*GSTP 1* glutathione S-transferase pi 1, *TLE3* transducin-like enhancer of split 3, *HER2* human epidermal growth factor receptor 2, *TNBC* triple-negative breast cancer

Based on a univariate analysis of 22 TNBC cases, a better PFS correlated significantly with a positive TLE3 expression in the tumor (*p* = 0.025). A multivariate logistic regression analysis by Ki67 and TLE3 including 22 patients with TNBC also showed that a positive TLE3 expression significantly correlated with a better PFS (*p* = 0.037, Hazard ratio = 0.126, 95% CI = 0.018–0.885). Therefore, TLE3 expression in the tumor was identified to be an independent predictive marker of the therapeutic effect of eribulin chemotherapy among patients with TNBC lesions (Table 5).

**Table 5:** Univariate and multivariate analysis with respect to progression free survival in 22 triple-negative breast cancers

Parameters	Univarite analysis			Multivariate analysis		
	Hazard ratio	95% CI	*p* value	Hazard ratio	95% CI	*p* value
Age at operation						
≤ 63 vs >63	0.787	0.249–2.489	0.683			
Degree of progress						
Locally advanced vs Visceral metastases	0.509	0.136–1.909	0.317			
Life threatening condition						
non- Life threatening vs Life threatening	1.368	0.403–4.647	0.615			
Nuclear grade						
1, 2, vs 3	0.534	0.154–1.849	0.322			
Ki67						
Negative vs Positive	0.295	0.083–1.052	0.060	0.190	0.024–1.482	0.113
GSTP1						
Negative vs Positive	1.514	0.402–5.709	0.540			
β-tubulin class III						
Negative vs Positive	0.548	0.147–2.046	0.371			
TLE3						
Negative vs Positive	0.148	0.028–0.788	0.025	0.126	0.018–0.885	0.037

*GSTP 1* glutathione S-transferase pi 1, *TLE3* transducin-like enhancer of split 3, *CI* confidence intervals

## Discussion

Eribulin is a synthetic derivative of Halichondrin B that was isolated from the sea sponge *Halichondria okadai* and is a new anticancer drug that is primarily composed of eribulin mesylate [30]. It exhibits an anticancer effect as a tubulin polymerization inhibitor by suppressing the extension of microtubules, thereby preventing normal spindle formation, stopping cell division and inducing apoptosis [12, 13]. In the present study, the ORR after eribulin treatment was 34.6%. This level of efficacy is relatively high compared with that observed in the main clinical trials [14, 31]. Such high efficacy may be due to the process of patient selection in our series, as we used eribulin in relatively earlier lines than those employed in the trials mentioned above. We found that eribulin achieves a higher response rate when used in front to earlier lines compared to when it is used in later lines, such as after more than three regimens with therapeutic failure, which is a common clinical application (data not shown) [32]. Moreover, results obtained in recent years have indicated a greater potential benefit with eribulin treatment against TNBC lesions compared with that noted in patients with non-TNBC lesions. Although no significant differences in efficacy were observed in this study when tumors were stratified according to the intrinsic subtype, more studies are necessary to determine differences in the efficacy of eribulin according to differences in the intrinsic subtype. At any rate, there is a high possibility that eribulin will be applied as a key drug in the future treatment of TNBC, and it is expected that the ability to predict the therapeutic effect will become critical.

In the present study, biomarkers, such as TLE3 [16–18], β-tubulin class III [20–22] and GSTP1 [23], which have previously been reported to be possible indicators of the efficacy of taxane drugs, were investigated for their ability to predict the therapeutic effect of eribulin. The TLE3 gene is a member of the Notch signal transduction pathway, which inhibits transcriptional activation, and, although the TLE3 gene product does not directly interact with DNA, TLE3 affects the regulatory region of the target gene via DNA binding with the transcription factor. The TLE3 expression has also been reported to be involved in the therapeutic effect of taxane. Taxane drugs bind to the β-tubulin in a microtubule, which is a polymer configured from a heterodimer resulting from the binding of α-tubulin and β-tubulin. It has been demonstrated that the expression level of β-tubulin class III is associated with the therapeutic effect of taxane, and it has been reported that the effect of taxane is attenuated in breast cancer patients with a high expression of β-tubulin class III in cancer tissues [22]. The Glutathione S-transferase (GST) family consists of enzymes that detoxify and neutralize electrophiles by bonding with reduced glutathione (GSH). Human GSTP1 has also recently attracted attention as a cancer marker due to its presence in many cancer cells, and correlation with malignancy and treatment resistance has been reported [23]. Moreover, a correlation with the severity of peripheral nerve disorders has been suggested on GSTP1 codon 105 polymorphisms [33].

According to clinical observations, eribulin does not show cross-resistance with other taxanes or even demonstrate efficacy in treating taxane-resistant tumors [34–36]. Therefore, it is not surprising to find that β-tubulin class III and GSTP1 were not sufficient markers for predicting the therapeutic effect of eribulin in our series. In contrast, we found that a positive TLE3 expression in TNBC lesions was useful as a molecular marker for predicting the therapeutic effect of eribulin.

As mentioned above, TLE3 genes are members of the Notch signal transduction pathway. Notch is a transmembrane protein receptor that transmits signals inside cells following stimulation by a ligand such as Delta/Jagged that is also a transmembrane protein [37, 38]. Notch is involved in the maintenance of the stemness of stem cells; therefore, its role in the maintenance of cancer stem cells has also recently attracted attention [39]. To date, the significance of Notch signal involvement in cancer stem cells has been reported in some cancers including brain tumors and breast cancer.

A correlation between TNBC and cancer stem cells has also been indicated [40]; therefore, the protein expression of TLE genes that are related to suppression of the Notch signal transduction pathway is thought to occur more commonly and distinctly in TNBC lesions than in non-TNBC lesions. Hence, meaningful involvement of TLE3 expression in eribulin chemosensitivity is observed only in cases of TNBC. Moreover, although involvement of the epithelial-mesenchymal transition (EMT) in the pathogenesis of TNBC is becoming increasingly clear, since recent reports have indicated that eribulin plays a role in EMT suppression [41], prediction of the therapeutic effect of eribulin may become possible by confirming TLE expression in TNBC lesions.

## Conclusions

Our findings suggest that TLE3 is a useful marker for predicting the therapeutic effect of eribulin chemotherapy for TNBC.

## Additional file

Additional file 1: Univariate and multivariate analysis with respect to progression free survival in 52 locally advanced or metastatic breast cancer. In a multivariate analysis including TLE3 and Ki67, no biomarkers useful for predicting the efficacy of eribulin in cases of MBC were found. (DOCX 16 kb)

## Abbreviations

17 GST: Glutathione S-transferase
CBR: Clinical benefit rate
CEP: Centromere
CR: Complete Response
DCR: Disease control rate
ER: Estrogen receptor
eribulin: eribulin mesylate
FISH: Fluorescent in situ hybridization
GSH: Glutathione
GSTP: Glutathione S-transferase pi
HE: Hematoxylin and eosin
HER: Human epidermal growth factor receptor
MBC: Locally advanced or metastatic breast cancer
NE: Not evaluable
ORR: overall response rate
OS: Overall survival
PD: Progressive disease
PFS: Progression-free survival
PgR: Progesterone receptor
PR: Partial Response
QOL: quality of life
RECIST: Response evaluation criteria in solid tumors
SD: Stable disease
TLE: transducin-like enhancer of split
TNBC: Triple-negative breast cancer
TTF: Time to treatment failure
