# Supplementary material for: Identification of predictive markers of the therapeutic effect of eribulin chemotherapy for locally advanced or metastatic breast cancer
Source: BMC Cancer. 2017 Aug 31;17:604. doi: 10.1186/s12885-017-3598-5 (PMC5580315; doi:10.1186/s12885-017-3598-5)
Supplement: Additional file 1: — Univariate and multivariate analysis with respect to progression free survival in 52 locally advanced or metastatic breast cancer. In a multivariate analysis including TLE3 and Ki67, no biomarkers useful for predicting the efficacy of eribulin in cases of MBC were found. (DOCX 16 kb) [file 12885_2017_3598_MOESM1_ESM.docx]

**Additional file 1. Univariate and multivariate analysis with respect to progression free survival in 52 locally advanced or metastatic breast cancer.**

|  | Univarite analysis | | |  | Multivariate analysis | | |
| --- | --- | --- | --- | --- | --- | --- | --- |
| Parameters | Hazard ratio | 95% CI | *p* value |  | Hazard ratio | 95% CI | *p* value |
| Age at operation  ≤63 vs >63 | 1.235 | 0.631-2.417 | 0.539 |  |  |  |  |
| Degree of progress  Locally advanced vs Visceral metastases | 0.597 | 0.284-1.255 | 0.174 |  |  |  |  |
| Life threatening condition  non- Life threatening vs Life threatening | 1.070 | 0.480-2.386 | 0.868 |  |  |  |  |
| Estrogen receptor  Negative vs Positive | 1.786 | 0.885-3.607 | 0.106 |  |  |  |  |
| Progesterone receptor  Negative vs Positive | 1.506 | 0.767-2.955 | 0.234 |  |  |  |  |
| HER2  Negative vs Positive | 0.602 | 0.182-1.990 | 0.405 |  |  |  |  |
| HR and HER2 status  TNBC vs non-TNBC | 0.715 | 0.353-1.448 | 0.352 |  |  |  |  |
| Nuclear grade  1, 2, vs 3 | 0.768 | 0.367-1.608 | 0.484 |  |  |  |  |
| Ki67  Negative vs Positive | 0.598 | 0.306-1.171 | 0.134 |  | 0.510 | 0.212-1.230 | 0.134 |
| GSTP1  Negative vs Positive | 1.095 | 0.557-2.153 | 0.792 |  |  |  |  |
| β-tubulin class III  Negative vs Positive | 1.473 | 0.744-2.916 | 0.267 |  |  |  |  |
| TLE3  Negative vs Positive | 0.766 | 0.388-1.515 | 0.444 |  | 1.098 | 0.504-2.394 | 0.814 |

TLE3, transducin-like enhancer of split 3. GSTP 1, glutathione S-transferase pi 1. HR, hormone receptor. HER2, human epidermal growth factor receptor 2. TNBC, triple-negative breast cancer. CI, confidence intervals.
